# Physical Activity, Self-Administered Motor Tests, and Academic Achievement in Lower Secondary School Students: A Cross-Sectional Study

**DOI:** 10.3390/children13091255

**Published:** 2026-09-16

**Authors:** Tiziana D’Isanto, Felice Di Domenico, Vera Simões, Giovanni Esposito, Sara Aliberti

**Affiliations:** 1 Research Center of Physical Education and Exercise, University of Pegaso, 80143 Naples, Italy; tizidisanto@gmail.com (T.D.); flcdidomenico@gmail.com (F.D.D.); sara.aliberti@unipegaso.it (S.A.); 2 School of Sport Rio Maior (ESDRM), Santarém Polytechnic University—Santarém & Rio Maior, 2040-413 Santarém, Portugal; verasimoes@esdrm.ipsantarem.pt

**Keywords:** academic achievement, physical activity, skill-related fitness, choice reaction performance, physical literacy, lower secondary school, motor competence, self-administered motor tests

## Abstract

**Background**: Physical activity is widely recognized as an important determinant of health and cognitive development; however, the association between specific components of motor performance and academic achievement remains incompletely understood. This cross-sectional study investigated the relationships between self-reported physical activity, self-administered motor tests, and self-reported academic achievement in lower secondary school students. **Methods**: A cross-sectional observational study was conducted involving 113 lower secondary school students (mean age: 12 years; 66 males and 47 females). Self-reported weekly physical activity and self-reported Grade Point Average (GPA) were collected using an online questionnaire. Following standardized familiarization during physical education classes, students self-administered the Stork Balance Stand Test, Plate Tapping Test, and Illinois Agility Test, whereas choice reaction performance was assessed individually under standardized conditions using the FITLIGHT Trainer system. Pearson’s correlation analyses, one-way ANOVA, and multiple linear regression were performed. **Results**: Weekly physical activity showed a strong positive correlation with GPA (r = 0.654, FDR-adjusted *p* < 0.001) and significant FDR-adjusted associations with upper-limb speed and choice reaction performance, whereas its associations with balance and agility did not remain statistically significant after correction for multiple comparisons. In the multiple regression analysis, weekly physical activity was the only variable independently associated with GPA (β = 0.639, *p* < 0.001), whereas balance, upper-limb speed, choice reaction performance, and agility were not independently associated with academic achievement after adjustment for age, sex, and school grade. The regression model explained 57.3% of the variance in GPA (adjusted *R*^2^ = 0.535; *p* < 0.001). **Conclusions**: In this sample of lower secondary school students, self-reported weekly physical activity was independently associated with self-reported academic achievement, whereas the assessed motor performance measures were not independently associated with GPA after adjustment for demographic variables. Given the cross-sectional design and reliance on self-reported measures, these findings should be interpreted as associative rather than causal. Further longitudinal studies using objective measures of physical activity and validated school-based motor assessment protocols are warranted.

## 1. Introduction

Physical activity during childhood and adolescence is widely recognized as a key determinant of physical and psychological health, contributing to healthy growth and the prevention of numerous chronic conditions and functional impairments [1,2]. In recent years, Exercise and Sport Sciences have progressively evolved into an interdisciplinary scientific field, emphasizing evidence-based approaches for the assessment, promotion, and prescription of physical activity in educational, clinical, and sport settings [3]. Within this framework, increasing attention has been devoted to understanding the influence of physical activity on cognitive development during childhood and adolescence [4,5]. A growing body of evidence indicates that regular physical activity positively influences attention, memory, executive functions, mood, and emotional well-being [6,7,8]. These beneficial effects have been documented across different population groups, including older adults, healthy adults, children, adolescents, and individuals with attention-deficit/hyperactivity disorder (ADHD) [9,10,11].

Within this framework, research has progressively shifted from a purely quantitative perspective, focused on the overall volume of physical activity, to a more comprehensive approach that also considers the qualitative characteristics of motor performance [11,12]. A substantial body of experimental and clinical evidence has demonstrated that physical activity promotes cognitive development through multiple neurobiological mechanisms, including increased cerebral blood flow, modulation of neurotransmitter systems, enhanced expression of neurotrophic factors, and improved neuroplasticity, ultimately leading to positive effects on executive functions, attention, and memory [13,14,15]. These mechanisms are particularly relevant during school age, a developmental period characterized by high brain plasticity and increased sensitivity to environmental stimuli [16,17]. In this context, movement should be regarded not only as a health-promoting behaviour but also as an educational resource capable of fostering cognitive, social, and transversal competencies that support learning processes and, consequently, academic achievement. Previous studies have shown that structured motor experiences and student-centered teaching approaches enhance not only motor competence but also executive functions, cooperation, problem-solving skills, and self-regulation, all of which are considered essential determinants of educational success [18,19]. In particular, active pedagogical approaches in physical education, such as heuristic learning, have proven effective in promoting teamwork, collaborative skills, and other transversal competencies that are fundamental to students’ educational development [20]. Within this perspective, specific components of motor efficiency, including balance, speed, agility, and choice reaction performance, have received increasing attention because of their potential relationship with cognitive functioning [21,22,23]. These variables represent partially distinct yet interconnected domains of neuromotor control. Balance primarily reflects postural regulation and sensory integration processes, which are closely related to attentional mechanisms. Speed and agility involve complex coordinative patterns, motor planning, and rapid execution, requiring higher-order cognitive processes, particularly executive functions. Choice reaction performance, in turn, represents a behavioural indicator of information-processing efficiency, encompassing stimulus detection, response selection, and motor execution.

The motor components investigated in the present study can be framed within the construct of skill-related fitness, which includes agility, balance, coordination, power, speed, and reaction-related abilities, and differs from health-related fitness, which primarily refers to cardiorespiratory fitness, muscular fitness, flexibility, and body composition [24,25]. Although these components have traditionally been associated with motor and sport performance, increasing evidence suggests that several neuromotor abilities are also related to cognitive functioning, particularly during childhood and adolescence [26,27]. Previous studies have reported associations between motor competence, agility, movement speed, coordination, and cognitive tasks involving information-processing speed and inhibitory control [28,29]. However, these relationships are not consistent across all motor domains, suggesting that different motor abilities may contribute differently to cognitive functioning and academic achievement. The selection of the variables included in this study is based on the hypothesis that specific components of motor efficiency may represent indirect indicators of cognitive processes underlying academic achievement. In particular, abilities such as balance, agility, speed, and choice reaction performance require efficient integration of perceptual information, motor planning, executive control, and rapid information processing, all of which have been associated with cognitive performance and learning processes [30,31].

Another relevant variable for a comprehensive interpretation of this relationship is the amount of weekly physical activity, which complements specific measures of motor performance by providing an overall behavioural indicator of habitual movement. Distinguishing between habitual physical activity and specific dimensions of motor performance may therefore help explain some of the inconsistencies reported in the previous literature. Although a growing body of evidence supports a positive relationship between physical activity and academic achievement, the contribution of specific components of motor performance remains less clearly understood. Previous studies have reported associations between motor competence, neuromotor abilities, cognitive functioning, and academic outcomes; however, these relationships appear to vary across motor domains. In particular, it remains unclear whether specific components of skill-related fitness provide additional information regarding academic achievement beyond that accounted for by habitual physical activity and demographic characteristics. Examining habitual physical activity together with distinct motor performance domains may therefore provide a more comprehensive understanding of their respective relationships with academic achievement in school-aged populations.

From a methodological perspective, school-based research examining physical activity and academic performance has predominantly relied on structured assessments conducted within supervised educational settings [32]. Self-administered motor tests conducted after standardized familiarization may represent a practical approach for school-based data collection; however, their reliability, validity, and feasibility require specific investigation. Accordingly, in the present study, self-administration was adopted as a methodological approach for selected motor tests rather than evaluated as an outcome itself. Importantly, this study does not aim to establish the reliability, validity, or agreement of self-administered motor testing compared with professionally administered protocols. Instead, self-administration was used to explore whether standardized motor assessments could be implemented within a school context after appropriate familiarization. Therefore, the findings should be interpreted exclusively in relation to the observed associations between motor performance variables and academic achievement, while formal validation studies remain necessary.

Within this framework, the present study aimed to examine the relationships between habitual physical activity, specific components of motor performance, and academic achievement among lower secondary school students. Specifically, the primary aim was to determine whether weekly physical activity, balance, upper-limb speed, agility, and choice reaction performance were independently associated with Grade Point Average (GPA) after accounting for demographic characteristics. As a secondary exploratory aim, differences in academic achievement and weekly physical activity across school grade levels were also examined. A methodological characteristic of the study was the implementation of selected self-administered motor tests following standardized familiarization procedures within the school setting. Based on the existing literature, we hypothesized that higher levels of weekly physical activity and better motor performance would be associated with higher academic achievement, as reflected by GPA. Specifically, positive associations were expected for balance and choice reaction performance, whereas faster performance in the timed upper-limb speed and agility tests was expected to be associated with higher GPA.

## 2. Materials and Methods

### 2.1. Study Design

A cross-sectional observational study was conducted to investigate the relationship between physical activity, motor performance, and academic achievement among lower secondary school students. The study was designed to examine both the associations between these variables and the independent contribution of specific components of skill-related fitness to academic achievement. Data on weekly physical activity, motor performance, and Grade Point Average (GPA) were collected simultaneously, allowing the examination of associations between these variables through correlational and multivariable analyses. The study is reported in accordance with the Strengthening the Reporting of Observational Studies in Epidemiology (STROBE) guidelines for cross-sectional studies [33].

### 2.2. Participants

A total of 138 lower secondary school students were initially assessed for eligibility. Participants were recruited from a public lower secondary school located in Fisciano (Salerno, Italy). Participation was voluntary. Participants were eligible if they were aged between 10 and 14 years, were enrolled in the participating lower secondary school, were eligible to participate in regular physical education activities, and had no cardiorespiratory, functional, or other health conditions preventing safe participation in physical education or motor testing. The inclusion and exclusion criteria are summarized in Table 1.

Of the 138 students initially assessed, 25 were excluded because they did not meet the eligibility requirements, declined participation, or had incomplete data for key study variables. The final analytical sample therefore consisted of 113 students (66 males and 47 females; mean age: 12.28 ± 1.05 years; age range: 10–14 years), including 38 first-grade, 44 second-grade, and 31 third-grade students. Data collected through the online questionnaire were recorded anonymously. The main demographic characteristics of the final analytical sample are presented in Table 2.

Both students and their parents or legal guardians received detailed information regarding the aims and procedures of the study. Written informed consent was obtained from the parents or legal guardians of all participants prior to data collection, and students provided their assent to participate.

No a priori sample-size calculation was performed, as the study involved the available population of eligible students within the participating school. The final analytical sample therefore reflected the number of participants who met the eligibility criteria and provided complete data for the variables included in the analyses.

### 2.3. Procedures and Instruments

Data were collected using a structured questionnaire administered through Google Forms, which included information on academic achievement, expressed as Grade Point Average (GPA), weekly physical activity, and motor assessment results. Before data collection, students underwent a familiarization period during regular physical education classes, during which they received standardized instructions and practical training on the correct execution and scoring procedures of the motor tests.

Following this familiarization, the Stork Balance Stand Test, Plate Tapping Test, and Illinois Agility Test were self-administered by the students according to standardized procedures, and the obtained results were subsequently entered by the participants into the online questionnaire. In the present study, the term self-administered therefore refers to tests that students were able to perform and report independently after receiving prior standardized instruction and practical familiarization, without individual administration by a researcher during data collection.

Choice reaction performance represented an exception to this procedure. Because the FITLIGHT Trainer system was available as a single unit and could not be provided to each participant, this assessment was performed individually in the school gym using the FITLIGHT Trainer system under standardized conditions. Accordingly, the choice reaction task was not considered a self-administered assessment.

Weekly physical activity was assessed using a structured domain-based self-report procedure specifically designed to estimate the total amount of physical activity accumulated across different contexts during the previous 7 days. Students reported the frequency and duration of activities performed in eight domains: (1) walking from home to school (number of trips and minutes per trip); (2) walking from school to home (number of trips and minutes per trip); (3) gym-based physical activity or training (number of sessions and minutes per session); (4) organized sport or physical activities, such as football, basketball, volleyball, dance, swimming, or martial arts (number of sessions and minutes per session); (5) active outdoor play, such as running, chasing games, or ball games (number of days and approximate minutes per day); (6) cycling (number of days and approximate minutes per day); (7) walking other than the home–school commute (number of days and approximate minutes per day); and (8) school physical education (number of lessons in which students actively participated and minutes per lesson).

For each domain, weekly physical activity was calculated by multiplying the reported frequency by the reported duration. Domain-specific estimates were then summed to obtain total weekly physical activity, expressed as minutes per week. Walking reported under the additional walking item explicitly excluded the home–school commute in order to avoid double counting. Activities were assigned to their corresponding domain and included only once in the calculation of total weekly physical activity.

No minimum duration or intensity threshold was applied. All reported activities falling within the predefined domains were included in the weekly estimate, as the purpose of the assessment was to estimate the overall volume of habitual physical activity rather than specifically moderate-to-vigorous physical activity (MVPA). Accordingly, the resulting variable should be interpreted as total self-reported physical activity accumulated during the previous 7 days across active transport, school physical education, structured exercise or sport, and recreational activities.

Academic achievement was assessed using students’ self-reported Grade Point Average (GPA). Data collection was conducted in December, and participants were instructed to report the overall GPA displayed in their electronic school record at the time of data collection rather than calculating the average themselves. Thus, the reported GPA reflected the academic grades recorded during the school year up to the time of assessment. GPA was expressed according to the Italian 10-point grading scale, in which 6 represents the minimum passing grade and 10 the highest attainable grade. Although the reported GPA was derived from the students’ electronic school records, it was entered directly by the participants into the online questionnaire and was not independently verified by the research team against official school records. Accordingly, GPA was treated as a self-reported measure in the present study.

Static balance was assessed using the Stork Balance Stand Test. Participants stood barefoot with their hands placed on their hips and positioned the non-supporting foot against the medial aspect of the knee, at the level of the medial femoral epicondyle of the supporting limb. They then raised the heel of the supporting foot and maintained balance on the forefoot for as long as possible. Timing was stopped when the participant lost the required position, including when the heel of the supporting foot touched the floor, the non-supporting foot moved away from the knee, the hands left the hips, or balance was otherwise lost. The test was performed separately on both limbs, and balance time was recorded in seconds. The mean of the values obtained for the right and left limbs was used for the statistical analyses.

Upper-limb speed was assessed using the Plate Tapping Test. Two circular discs (20 cm in diameter) were positioned on a table with their centers 80 cm apart, resulting in a distance of 60 cm between their nearest edges. A rectangular plate (10 cm × 20 cm) was positioned midway between the two discs. Participants performed the test using a standard household table and stood facing the testing surface with their feet slightly apart. The non-dominant hand was placed flat on the central rectangular plate and remained in this position throughout the test, while the dominant hand was initially placed on one of the two discs. At the start signal, participants moved the dominant hand back and forth as quickly as possible, alternately touching the two discs for 25 complete cycles (50 touches in total). Performance was recorded as the time, in seconds, required to complete the 25 cycles. Two trials were performed, and the fastest time was retained for the statistical analyses.

Agility was assessed using the Illinois Agility Test. The testing course was 10 m long and 5 m wide. Four cones were used to mark the start, finish, and two turning points, while four additional cones were positioned along the center of the course at equal intervals of approximately 3.3 m. Participants started in a prone position at the starting line. To allow self-administration of the test, timing was performed using a stopwatch application on a smartphone set with a 20 s delayed start. The smartphone was positioned on the right side of the finish cone, allowing the participant sufficient time to assume the starting position before the timing began. At the start signal, participants stood up as quickly as possible and completed the prescribed course, including the changes in direction and slalom sections, without displacing the cones. Upon reaching the finish line, participants manually stopped the stopwatch application positioned adjacent to the finish cone. Performance was recorded in seconds, with lower values indicating better agility performance. Although previous studies have demonstrated good-to-excellent test–retest reliability of the Illinois Agility Test when administered under standardized conditions [34], to our knowledge, the specific self-administered and self-timed smartphone procedure adopted in the present study has not previously been validated. Nevertheless, self-administered physical performance assessments supported by smartphone technology have been investigated for other field-based tests. For example, a self-administered smartphone-based Six-Minute Walk Test has shown excellent test–retest reliability and reproducibility [35]. These findings support the methodological feasibility of smartphone-assisted self-administration in other physical performance testing contexts but cannot be generalized to the Illinois Agility Test protocol used in the present study. Therefore, the reliability and validity of the specific self-timed procedure adopted here should be considered unestablished.

Choice reaction performance was assessed using the FITLIGHT Trainer™ System (FTS; Fitlight Sports Corp., Aurora, ON, Canada) under controlled testing conditions and was administered individually by the researchers. Four light-emitting sensor discs were mounted on a wall in a square configuration, with approximately 1.5 m between adjacent discs and positioned at approximately hand-to-shoulder height. Participants stood facing the center of the configuration at a distance of approximately 50 cm from the wall. During the 30 s task, the lights were activated individually in a random sequence, and participants were instructed to deactivate each illuminated sensor as quickly as possible by touching it. Performance was quantified as the number of successfully deactivated lights (hits) during the 30 s testing period, with higher values indicating better task-specific response performance. Before data collection, participants completed a familiarization trial. Two test trials were subsequently performed, and the mean number of successfully deactivated lights across the two trials was used for statistical analyses. Previous research has reported adequate test–retest reliability and known-groups validity for visuomotor response protocols performed using the FITLIGHT Trainer™ System [36]. However, because measurement properties are protocol-specific, these findings cannot be assumed to apply directly to the specific four-disc, 30 s configuration employed in the present study. The reliability and criterion validity of the present testing configuration were not independently evaluated. Therefore, choice reaction results should be interpreted as performance indicators derived from a standardized device-based task rather than as a direct measure of cognitive function.

### 2.4. Ethics Statement

The present study was conducted as part of the research project entitled “Psychophysical Perception and Body Awareness in School and Sports Contexts through Observational and Non-Invasive Tools” and was approved by the Ethics Committee of Pegaso University (Prot./E 004726, approved on 15 July 2025). The study was conducted in accordance with the Declaration of Helsinki. Written informed consent was obtained from the parents or legal guardians of all participants prior to data collection.

### 2.5. Statistical Analysis

Statistical analyses were performed using JASP (version 0.97.1; University of Amsterdam, Amsterdam, The Netherlands). Continuous variables are presented as mean ± standard deviation (SD), with median and interquartile range (IQR) additionally reported to provide distributional information, whereas categorical variables are presented as frequencies and percentages. The distribution of continuous variables was assessed through visual inspection of histograms and Q–Q plots and by applying the Shapiro–Wilk test. Homogeneity of variances was evaluated using Levene’s test before between-group comparisons. Pearson’s correlation coefficients were calculated to examine the associations among GPA, weekly physical activity, and motor performance variables. To account for multiple testing across the 15 pairwise correlations, the Benjamini–Hochberg procedure was applied to control the false discovery rate (FDR) at 5%. Accordingly, statistical significance for the correlation analyses was determined using FDR-adjusted *p*-values, with an adjusted *p*-value < 0.05 considered statistically significant. Differences between sexes were assessed using independent-samples *t*-tests, whereas one-way analysis of variance (ANOVA) with Bonferroni-adjusted post hoc comparisons was used to compare variables across school grades. Effect sizes for one-way ANOVA were reported as eta squared (η^2^). Multiple linear regression analysis was performed to identify variables independently associated with GPA. Prior to model estimation, multicollinearity was evaluated using variance inflation factors (VIFs), while residual plots were visually inspected to assess linearity and homoscedasticity. Standardized regression coefficients (β), 95% confidence intervals (95% CI), and model fit indices are reported. Statistical significance was set at *p* < 0.05.

## 3. Results

### 3.1. Participant Flow

**Figure 1:**
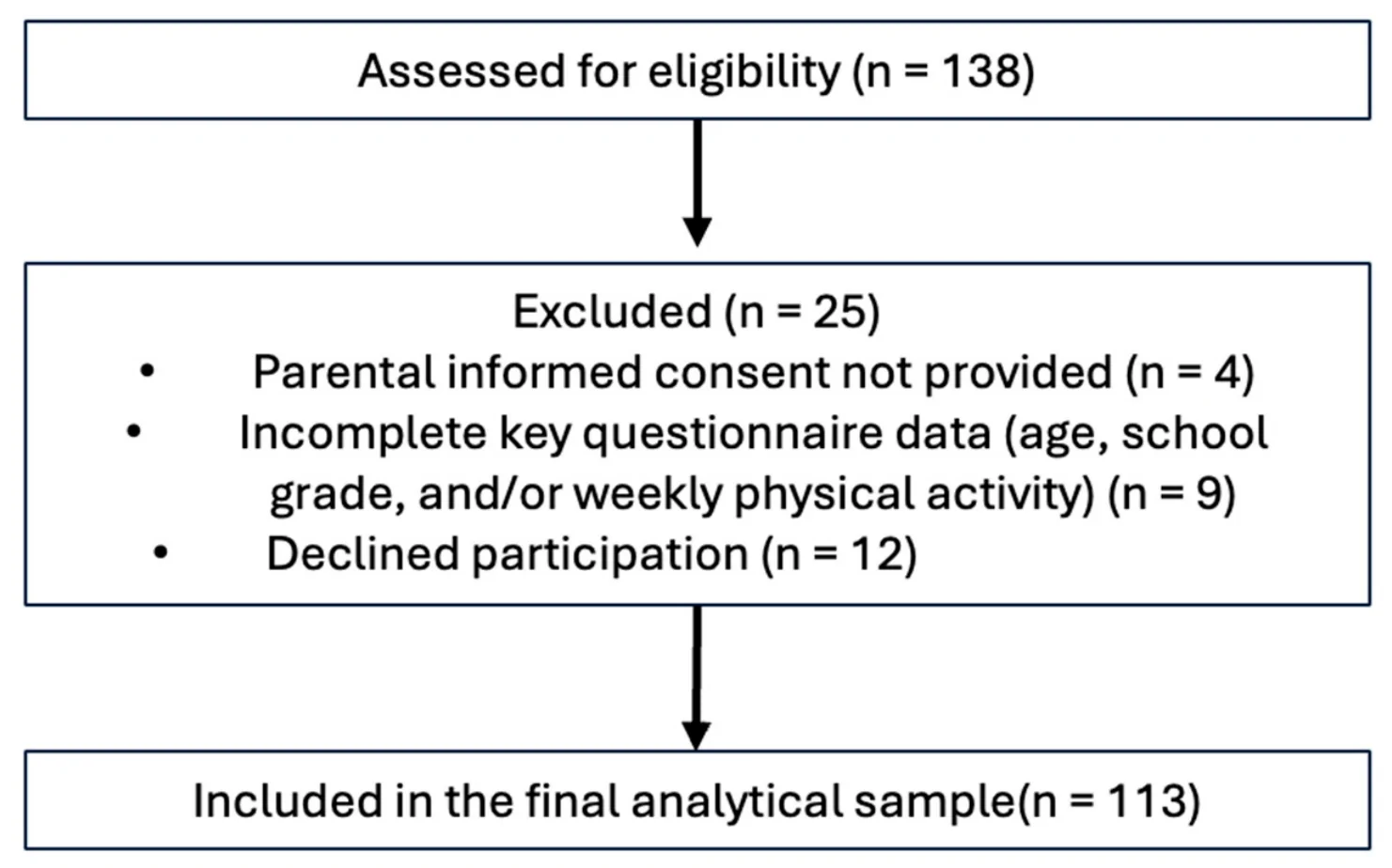
Flow diagram of participant recruitment and inclusion in the final analytical sample.

Figure 1 summarizes the participant recruitment process. A total of 138 lower secondary school students were initially assessed for eligibility. Of these, 25 were excluded: 4 because parental informed consent was not provided, 9 because of incomplete data for key study variables (age, school grade, weekly physical activity, and/or GPA), and 12 because they declined participation. The final analytical sample therefore consisted of 113 students.

### 3.2. Descriptive Statistics

Descriptive statistics for all study variables are presented in Table 3. The study sample (N = 113) accumulated an average of 1034 ± 243.6 min/week of physical activity and achieved a mean Grade Point Average (GPA) of 8.25 ± 1.14, indicating an overall good level of academic achievement. Regarding motor performance, the mean values were 2.79 ± 1.74 s for the Stork Balance Stand Test, 21.89 ± 4.65 s for the Plate Tapping Test, 20.16 ± 2.45 for choice reaction performance, and 15.91 ± 2.35 s for the Illinois Agility Test.

Descriptive statistics by sex are presented in Table 4. No statistically significant differences were observed between males and females for any of the study variables (all *p* > 0.05), including weekly physical activity, academic achievement (GPA), and the motor performance measures.

No statistically significant differences were observed between males and females for the assessed variables. However, the absence of statistically significant differences should not be interpreted as evidence of equivalence between sexes; therefore, sex was included as a covariate in the regression model.

### 3.3. Correlation Analysis

Pearson correlation analyses showed a strong positive correlation between GPA and weekly physical activity (r = 0.654, FDR-adjusted *p* < 0.001). A weak positive correlation was observed between GPA and balance performance (r = 0.198, unadjusted *p* = 0.036); however, this association did not remain statistically significant after Benjamini–Hochberg correction (FDR-adjusted *p* = 0.067). GPA was not significantly correlated with upper-limb speed, choice reaction performance, or agility. Weekly physical activity was negatively correlated with Plate Tapping Test time (r = −0.276, FDR-adjusted *p* = 0.012) and positively correlated with choice reaction performance (r = 0.311, FDR-adjusted *p* = 0.004). Its weak positive correlation with balance (r = 0.206) and weak negative correlation with Illinois Agility Test time (r = −0.208) did not remain statistically significant after FDR correction (both FDR-adjusted *p* = 0.061). Balance performance was positively correlated with choice reaction performance (r = 0.230, FDR-adjusted *p* = 0.043), while Plate Tapping Test time was positively correlated with Illinois Agility Test time (r = 0.564, FDR-adjusted *p* < 0.001) (Table 5).

### 3.4. Multiple Regression Analysis of Academic Achievement

To examine the variables independently associated with academic achievement, a multiple linear regression analysis was performed including weekly physical activity, Stork Balance Stand Test, Plate Tapping Test, choice reaction performance, Illinois Agility Test, age, sex, and school grade as independent variables. The overall regression model (Table 6) was statistically significant (*F*(9, 103) = 15.34, *p* < 0.001) and explained 57.3% of the variance in self-reported GPA (*R*^2^ = 0.573; adjusted *R*^2^ = 0.535). Assumption testing indicated no evidence of problematic multicollinearity, with variance inflation factor (VIF) values ranging from 1.09 to 1.53. Among the variables included in the model, weekly physical activity was the strongest independent variable associated with GPA (β = 0.639, *B* = 0.00299, 95% CI: 0.00232 to 0.00367, *p* < 0.001). Age was negatively associated with GPA (β = −0.259, *B* = −0.282, 95% CI: −0.562 to −0.001, *p* = 0.049). Regarding school grade, students attending the second grade showed significantly lower GPA values than first-grade students (*B* = −0.489, 95% CI: −0.963 to −0.016, *p* = 0.043), whereas no significant difference was observed between third- and first-grade students (*p* = 0.558). The Stork Balance Stand Test, Plate Tapping Test, choice reaction performance, Illinois Agility Test, and sex were not independently associated with GPA after adjustment for the other variables included in the model (all *p* > 0.05).

### 3.5. Differences Across School Grades

One-way analysis of variance (ANOVA) was performed to examine differences in academic achievement and weekly physical activity across the three school grade levels. A significant effect of school grade level on Grade Point Average (GPA) was observed (*F*(2, 110) = 6.335, *p* = 0.002, η^2^ = 0.103). Bonferroni-adjusted post hoc comparisons showed that first-grade students had significantly higher GPA values than both second-grade students (mean difference = 0.79, *p* = 0.004) and third-grade students (mean difference = 0.75, *p* = 0.016). No significant difference was observed between second- and third-grade students (mean difference = −0.04, *p* = 1.000). In contrast, no statistically significant differences in weekly physical activity were observed across school grade levels (*F*(2, 110) = 0.010, *p* = 0.990, η^2^ < 0.001). The results of the one-way ANOVA are presented in Table 7.

**Figure 2:**
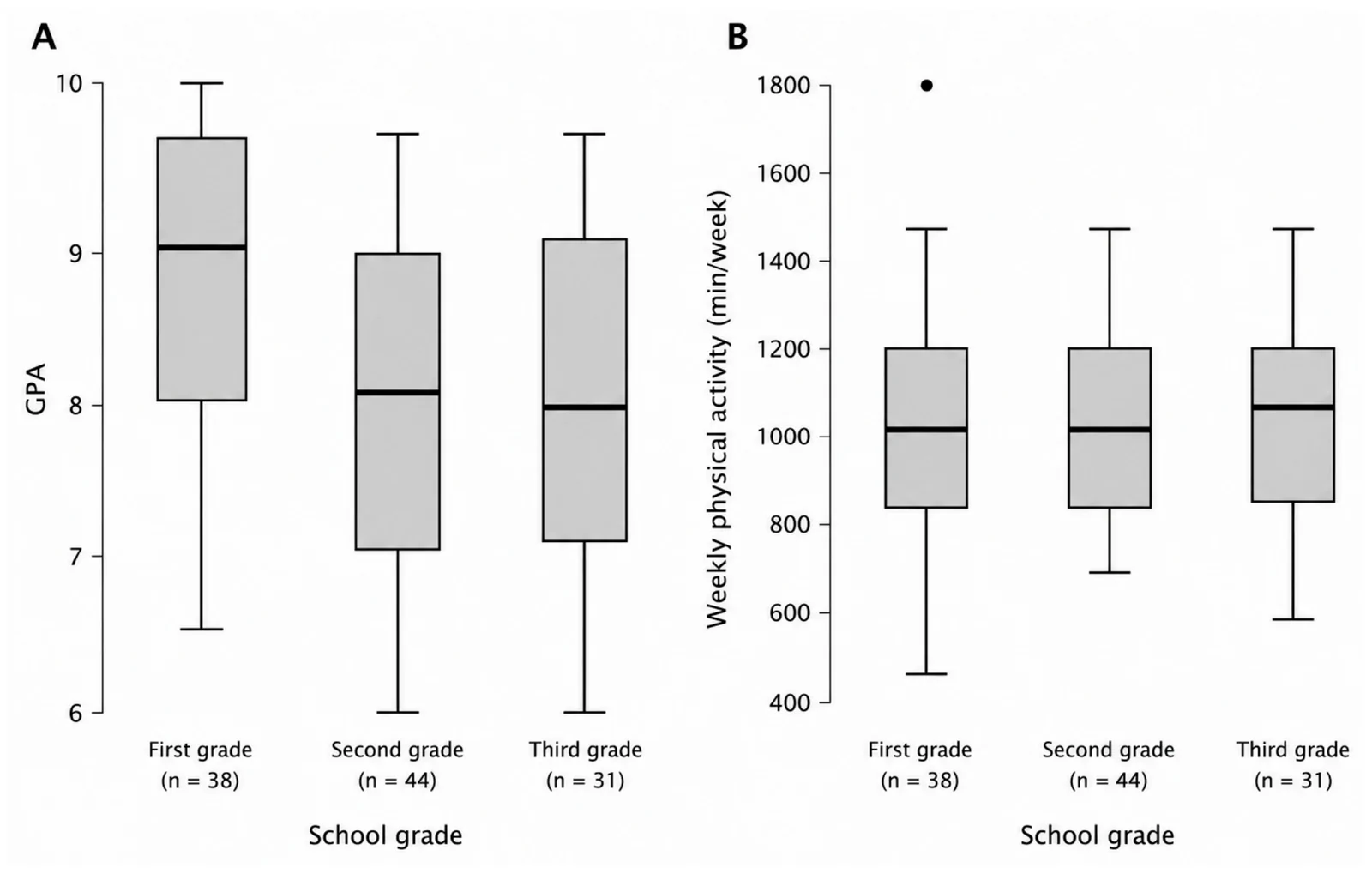
Distribution of academic achievement and weekly physical activity across school grade levels. (**A**) Boxplots of Grade Point Average (GPA) across first-, second-, and third-grade students. (**B**) Boxplots of weekly physical activity (min/week) across first-, second-, and third-grade students. Boxes represent the interquartile range (25th–75th percentiles), horizontal lines indicate the median, whiskers extend to the most extreme data points within 1.5 × IQR from the quartiles, and points represent outliers.

The distribution of academic achievement and weekly physical activity across school grade levels is illustrated in Figure 2.

## 4. Discussion

The main finding of the present study was the strong positive association between self-reported weekly physical activity and self-reported academic achievement among lower secondary school students. Weekly physical activity was strongly correlated with GPA and remained the strongest independently associated variable in the multivariable model after accounting for age, sex, school grade, and the assessed motor performance domains. In contrast, the contribution of specific motor performance components was less evident. Balance showed a weak positive bivariate correlation with GPA; however, this association did not remain statistically significant after correction for multiple comparisons. Upper-limb speed, choice reaction performance, and agility were not significantly correlated with academic achievement. Moreover, none of the assessed motor performance variables remained independently associated with GPA in the adjusted regression model. These findings suggest that, within the present sample, the overall amount of habitual physical activity was more consistently associated with academic achievement than performance in the specific skill-related fitness domains assessed.

The positive association observed between weekly physical activity and academic achievement is consistent with previous evidence demonstrating that regular exercise promotes cognitive development through multiple neurobiological mechanisms, including increased cerebral blood flow, modulation of neurotransmitter systems, enhanced expression of neurotrophic factors, and improved neuroplasticity [14,15,37,38]. Collectively, these adaptations have been associated with improvements in attention, working memory, executive functions, and other cognitive processes that support learning and academic performance. Nevertheless, given the cross-sectional design of the present study, these mechanisms should be regarded as plausible explanations of the observed associations rather than direct evidence of causality.

The present findings may also be interpreted within the broader framework of physical literacy, which emphasizes the integrated development of motor competence, motivation, confidence, and lifelong engagement in physical activity as essential components of overall human development [39,40,41]. However, because physical literacy was not directly assessed in the present study, this interpretation should be considered speculative. It is therefore possible that students who engage more regularly in physical activity may also exhibit higher levels of motivation, self-confidence, and school engagement, which may be associated with better academic achievement [42].

Although choice reaction performance showed a positive correlation with weekly physical activity, it did not remain independently associated with GPA in the multivariable regression model. Similarly, balance, upper-limb speed, and agility were not independently associated with academic achievement after adjustment for the other variables included in the model. These findings suggest that the relationships observed in the univariate analyses may be largely explained by the influence of habitual physical activity and demographic factors rather than by independent contributions of the assessed motor performance measures.

From a neurophysiological perspective, performance in a choice reaction task reflects the efficiency of information-processing mechanisms involved in stimulus identification, response selection, motor programming, and movement initiation [43]. These processes involve several higher-order cognitive functions, including selective attention, processing speed, executive control, and decision-making, which have been associated with academic performance in previous research [44]. However, because cognitive functions were not directly assessed in the present study, choice reaction performance should not be interpreted as a direct indicator of cognitive efficiency.

The positive association observed between choice reaction performance and weekly physical activity is consistent with previous studies reporting links between regular physical activity, neuromotor performance, reaction-related abilities, inhibitory control, and cognitive functioning in children and adolescents [29,45]. Nevertheless, the lack of a significant association with GPA in both the bivariate and multivariable analyses suggests that choice reaction performance, despite its association with weekly physical activity, was not directly associated with academic achievement in the present sample.

Similarly, balance, upper-limb speed, and agility were not independently associated with academic achievement after adjustment for the other variables included in the model. Although these motor abilities represent important components of skill-related fitness and share common neuromotor mechanisms related to postural regulation, motor coordination, and movement efficiency [46,47], the present findings indicate that they did not provide additional explanatory value for GPA beyond that accounted for by weekly physical activity and demographic variables. Overall, these results support the multidimensional nature of the relationship between motor competence and academic achievement while suggesting that the different components of skill-related fitness may contribute differently to this association.

One-way ANOVA revealed significant differences in academic achievement across school grades, with post hoc comparisons showing higher GPA values in first-grade students than in both second- and third-grade students, whereas no significant difference was observed between second- and third-grade students. In the adjusted multiple regression model, however, only the difference between second- and first-grade students remained statistically significant. Because the present study employed a cross-sectional design, these findings should not be interpreted as evidence of a progressive decline in academic achievement over time. Rather, they may reflect differences between cohorts or other educational and contextual factors not assessed in the present study. Weekly physical activity did not differ significantly among school grades, suggesting that the observed differences in GPA are unlikely to be explained solely by variations in habitual physical activity. In addition, sex was not significantly associated with GPA in the adjusted regression model, although no interaction analyses were performed. Therefore, the present findings do not allow conclusions regarding whether the observed associations differ between male and female students.

Another methodological feature of the present study was the self-administration of selected motor tests following standardized familiarization procedures. In the present study, this approach was used as a data-collection procedure rather than evaluated as an outcome itself. Because no formal indicators of feasibility, such as completion rates, administration time, participant burden, acceptability, or resource requirements, were collected, the present findings do not provide evidence regarding the feasibility, scalability, or cost-effectiveness of self-administered motor assessment in school settings. Dedicated studies are therefore required to evaluate these aspects before broader implementation can be considered.

Although the multiple regression model was statistically significant and explained 57.3% of the variance in academic achievement, GPA remains a multifactorial outcome influenced by numerous determinants that were not considered in the present study, including socioeconomic status, family environment, teaching quality, learning motivation, sleep quality, biological maturation, psychological well-being, and other individual characteristics. Therefore, the observed associations should be interpreted within the context of these unmeasured factors, and weekly physical activity should be regarded as one of several variables associated with academic achievement rather than its sole determinant.

Several methodological strengths of the present study should also be acknowledged. First, habitual physical activity and multiple components of skill-related motor performance were examined within the same analytical framework, allowing their respective associations with academic achievement to be considered simultaneously. Second, the motor assessments were conducted using standardized instructions and a structured familiarization period within the school setting, while choice reaction performance was assessed individually under standardized conditions using the FITLIGHT Trainer system. Third, the multivariable analysis accounted for relevant demographic characteristics, including age, sex, and school grade, and included assessment of multicollinearity and model assumptions. Finally, the distinction between self-reported measures, self-administered motor assessments, and researcher-administered testing was maintained throughout the study, supporting a transparent interpretation of the methodological scope of the findings.

Several limitations should be acknowledged when interpreting the present findings. First, the cross-sectional design precludes any causal inference regarding the observed associations among the investigated variables. Therefore, the directionality of the relationship between physical activity and academic achievement cannot be established, and longitudinal or experimental studies are needed to clarify the temporal and potentially causal nature of this association.

Second, both weekly physical activity and Grade Point Average (GPA) were obtained through self-report, which may introduce reporting bias. Furthermore, because both variables were reported by the same participants within the same data-collection session, the possibility of common method bias cannot be excluded and may have contributed to the magnitude of the observed association between weekly physical activity and GPA [48]. However, students were instructed to report the GPA value recorded in their electronic school records, reducing the likelihood of inaccurate recall for academic achievement. Weekly physical activity was assessed through a structured domain-based self-report approach rather than through a validated physical activity questionnaire or objective monitoring device. Moreover, the estimate represented total physical activity volume without applying intensity-specific thresholds and should therefore not be interpreted as a measure of moderate-to-vigorous physical activity (MVPA). Although this procedure allowed an estimation of habitual physical activity patterns, the resulting estimates should be interpreted with caution. Future studies should confirm these findings using validated questionnaires and/or objective measures such as accelerometry.

Third, although the Stork Balance Stand Test, Plate Tapping Test, and Illinois Agility Test are established field-based motor assessments, their specific self-administered application following familiarization has not been formally validated against researcher-administered assessments. The present study was not designed as a reliability study, and no dedicated test–retest assessment was conducted to quantify the reproducibility of the self-administered procedures. Consequently, potential measurement error associated with self-administration cannot be quantified and may have influenced the observed associations. This limitation is particularly relevant because self-administration represented a methodological feature of the present study. Future studies should therefore specifically evaluate the test–retest reliability and agreement of these procedures using appropriate reliability statistics, including intraclass correlation coefficients with 95% confidence intervals and measures of absolute measurement error.

Additional limitations include the relatively small sample size and recruitment from a single school, which may limit the generalizability of the findings to broader populations of lower secondary school students. In addition, the relatively limited sample size in relation to the number of predictors warrants caution regarding the precision and stability of individual regression coefficients. Replication in larger independent samples is therefore needed. Moreover, the analyses did not account for several potentially relevant confounding factors, including socioeconomic status, sleep quality, biological maturation, dietary habits, family environment, psychological well-being, and direct measures of cognitive functioning. The possibility of residual confounding should therefore be considered when interpreting the observed associations, and future research should incorporate these individual, family, and school-related factors.

Finally, no formal indicators of feasibility, such as administration time, completion rates, implementation costs, participant acceptability, or testing errors, were collected. Consequently, the feasibility and scalability of the proposed assessment protocol cannot be established from the present findings and should be specifically evaluated in future studies before the protocol is recommended for large-scale school-based implementation.

Future research should adopt longitudinal or experimental designs, recruit larger multicentre samples, and integrate objective assessments of physical activity, cognitive-related outcomes, and motor performance. Further studies are warranted to establish the reliability, validity, agreement, and measurement error of self-administered motor assessment procedures by comparing them with researcher-administered assessments performed under standardized conditions. Such studies should determine whether the measurement error associated with self-administered procedures is comparable to that observed under professionally supervised conditions, thereby improving the reproducibility and interpretation of school-based motor assessments. In addition, future investigations should examine the potential mediating and moderating roles of individual, family, and school-related factors in the relationship between physical activity, motor performance, and academic achievement.

From an applied perspective, the present findings support the relevance of promoting physical activity within school settings and suggest that simple neuromotor assessment tools may provide complementary information on students’ motor performance. If future studies establish the reliability, validity, and feasibility of self-administered motor tests relative to traditional assessment protocols, these instruments may have potential applications in school-based motor assessment. Although the present findings do not establish causal relationships, they reinforce the relevance of physical activity as an integral component of multidimensional school-based strategies aimed at promoting physical well-being and supporting students’ overall educational development.

## 5. Conclusions

The present cross-sectional study found that self-reported weekly physical activity was independently associated with self-reported academic achievement among lower secondary school students after adjustment for age, sex, school grade, and motor performance measures. Although several motor performance measures were associated with weekly physical activity, none remained independently associated with Grade Point Average (GPA) in the multivariable analysis.

These findings support the existence of an association between habitual physical activity and academic achievement while confirming that academic performance is influenced by multiple factors beyond the variables assessed in the present study. Therefore, the observed relationships should be interpreted as associative rather than causal.

The use of self-administered motor tests following standardized familiarization procedures was a methodological feature of the present study. However, because the reliability, validity, and feasibility of the specific self-administered procedures were not formally established, no conclusions can be drawn regarding their suitability for routine educational monitoring or large-scale implementation. Dedicated validation and feasibility studies are required before such applications can be recommended.

Future longitudinal and experimental studies incorporating objective measures of physical activity, validated motor assessments, and direct evaluations of cognitive function are warranted to clarify the mechanisms underlying the observed associations and to further investigate the role of physical activity in supporting academic achievement.

## Figures and Tables

**Table 1:** Inclusion and exclusion criteria for participant recruitment and analysis.

Inclusion Criteria	Exclusion Criteria
Age between 10 and 14 years	Age outside the predefined range of 10–14 years
Enrolment in the participating lower secondary school	Presence of cardiorespiratory, functional, or other health conditions preventing safe participation in physical education or motor testing
Eligibility to participate in regular physical education activities	Ineligibility to participate in regular physical education activities
Written informed consent from a parent or legal guardian	Absence of written informed consent from a parent or legal guardian
Student assent to participate	Student declining or withdrawing participation
Participation in the standardized familiarization procedures for the motor assessments	Failure to participate in the standardized familiarization procedures
Complete data for the key study variables, including age, school grade, weekly physical activity, and GPA	Incomplete data for one or more key study variables, including age, school grade, weekly physical activity, or GPA
Completion of all required motor assessments	Failure to complete one or more of the required motor assessments

**Table 2:** Demographic characteristics of the study sample.

Characteristic	Total Sample (N = 113)
Age (years), mean ± SD	12.28 ± 1.05
Age range (years)	10–14
Male, *n* (%)	66 (58.4)
Female, *n* (%)	47 (41.6)
First grade, *n* (%)	38 (33.6)
Second grade, *n* (%)	44 (38.9)
Third grade, *n* (%)	31 (27.4)

**Table 3:** Descriptive statistics of the study variables (N = 113).

Variable	Min	Max	Mean ± SD	Median (IQR)
Weekly physical activity (min/week)	480	1800	1034 ± 243.6	1020 (360)
GPA	6.00	10.00	8.25 ± 1.14	8.28 (1.64)
Stork Balance Stand Test (s)	0.93	12.14	2.79 ± 1.74	2.30 (1.60)
Plate Tapping Test (s)	10.93	33.00	21.89 ± 4.65	21.16 (7.30)
Choice reaction performance (hits)	12	26	20.16 ± 2.45	20.00 (3.00)
Illinois Agility Test (s)	12.00	23.92	15.91 ± 2.35	15.31 (2.72)

Note. Choice reaction performance represents the mean number of successfully deactivated lights (hits) across two 30 s trials; higher values indicate better performance.

**Table 4:** Descriptive statistics by sex and group comparisons.

Variable	Sex	N	Mean	SD	*p*-Value
Weekly physical activity (min/week)	M	66	1041.79	265.25	0.669
	F	47	1022.61	210.55	
Grade point average (GPA)	M	66	8.10	1.25	0.088
	F	47	8.47	0.94	
Stork Balance Stand Test (s)	M	66	2.76	1.74	0.804
	F	47	2.84	1.76	
Plate Tapping Test (s)	M	66	21.67	4.88	0.545
	F	47	22.21	4.34	
Choice reaction performance (FITLIGHT Trainer)	M	66	20.11	2.60	0.790
	F	47	20.23	2.26	
Illinois Agility Test (s)	M	66	15.94	2.40	0.883
	F	47	15.88	2.29	

Note. SD = standard deviation. *p*-values refer to comparisons between males and females.

**Table 5:** Pearson correlation matrix with Benjamini–Hochberg FDR-adjusted *p*-values.

Variable	1	2	3	4	5	6
1. Grade point average (GPA)	—					
2. Weekly physical activity	0.654 (<0.001)	—				
3. Stork Balance Stand Test	0.198 (0.067)	0.206 (0.061)	—			
4. Plate Tapping Test	−0.083 (0.478)	−0.276 (0.012)	0.047 (0.665)	—		
5. Choice reaction performance (FITLIGHT Trainer)	0.166 (0.124)	0.311 (0.004)	0.230 (0.043)	0.164 (0.124)	—	
6. Illinois Agility Test	−0.099 (0.405)	−0.208 (0.061)	−0.002 (0.983)	0.564 (<0.001)	−0.068 (0.547)	—

Note. Values are Pearson’s correlation coefficients (r), with Benjamini–Hochberg false discovery rate (FDR)-adjusted *p*-values in parentheses. The Benjamini–Hochberg procedure was applied across the 15 pairwise correlations. Bold values indicate correlations that remained statistically significant after FDR correction (pFDR < 0.05).

**Table 6:** Multiple linear regression analysis examining variables independently associated with self-reported Grade Point Average (GPA).

Variable	B	SE	β	t	*p*	95% CI	VIF
Intercept	7.938	1.997	—	3.974	<0.001	3.977 to 11.900	—
Weekly physical activity (min/week)	0.00299	0.00034	0.639	8.776	<0.001	0.00232 to 0.00367	1.527
Stork Balance Stand Test (s)	0.0635	0.0460	0.097	1.382	0.170	−0.0277 to 0.1547	1.090
Plate Tapping Test (s)	0.0185	0.0198	0.076	0.934	0.353	−0.0208 to 0.0578	1.256
Choice reaction performance (FITLIGHT Trainer)	0.0158	0.0354	0.034	0.446	0.657	−0.0544 to 0.0859	1.181
Illinois Agility Test (s)	0.0077	0.0428	0.016	0.179	0.858	−0.0772 to 0.0925	1.367
Age (years)	−0.282	0.141	−0.259	−1.994	0.049	−0.562 to −0.001	1.525
Sex	−0.055	0.159	—	−0.350	0.727	−0.370 to 0.259	1.067
School grade (second vs. first)	−0.489	0.239	—	−2.049	0.043	−0.963 to −0.016	1.509
School grade (third vs. first)	−0.238	0.405	—	−0.588	0.558	−1.042 to 0.565	1.530

Note. School grade was entered as a categorical variable, with first grade as the reference category. Sex was entered as a categorical variable. Model statistics: *R* = 0.757; *R*^2^ = 0.573; Adjusted *R*^2^ = 0.535; RMSE = 0.778; *F*(9, 103) = 15.34; *p* < 0.001. B = unstandardized regression coefficient; SE = standard error; β = standardized regression coefficient (reported only for continuous predictors); CI = confidence interval; VIF = variance inflation factor; RMSE = root mean square error.

**Table 7:** Analysis of variance for academic achievement and weekly physical activity across school grade levels.

Outcome Variable	Source	Sum of Squares	df	Mean Square	F	*p*	η^2^
GPA	School grade level	15.06	2	7.528	6.335	0.002	0.103
	Residuals	130.71	110	1.188			
Weekly physical activity (min/week)	School grade level	1234	2	617.0	0.010	0.990	<0.001
	Residuals	6.647 × 10^6^	110	6.043 × 10^4^			

Note. GPA = Grade Point Average.

## Data Availability

The data presented in this study are available on request from the corresponding author. The data are not publicly available due to privacy and ethical reasons.
